# Preparation and characterization of site-specific dechlorinating microbial inocula capable of complete dechlorination enriched in anaerobic microcosms amended with clay mineral

**DOI:** 10.1007/s11274-020-2806-7

**Published:** 2020-02-03

**Authors:** Zsuzsanna Nagymáté, Laura Jurecska, Csaba Romsics, Fanni Tóth, Viktória Bódai, Éva Mészáros, Attila Szabó, Balázs Erdélyi, Károly Márialigeti

**Affiliations:** 1 Department of Microbiology, ELTE - Eötvös Loránd University, Pázmány Péter sétány 1/C, 1117 Budapest, Hungary; 2 Fermentia Ltd, Berlini u. 47-49, 1045 Budapest, Hungary; 3 Institute of Agricultural Sciences, ETH Zurich, 8315 Lindau, Switzerland

**Keywords:** Dechlorination, *Dehalococcoides* sp., Enrichment, Bioaugmentation, Reductive dehalogenase genes

## Abstract

**Abstract:**

Short-chain halogenated aliphatic hydrocarbons (e.g. perchloroethene, trichloroethene) are among the most toxic environmental pollutants. Perchloroethene and trichloroethene can be dechlorinated to non-toxic ethene through reductive dechlorination by *Dehalococcoides* sp. Bioaugmentation, applying cultures containing organohalide-respiring microorganisms, is a possible technique to remediate sites contaminated with chlorinated ethenes. Application of site specific inocula is an efficient alternative solution. Our aim was to develop site specific dechlorinating microbial inocula by enriching microbial consortia from groundwater contaminated with trichloroethene using microcosm experiments containing clay mineral as solid phase. Our main goal was to develop fast and reliable method to produce large amount (100 L) of bioactive agent with anaerobic fermentation technology. Polyphasic approach has been applied to monitor the effectiveness of dechlorination during the transfer process from bench-scale (500 mL) to industrial-scale (100 L). Gas chromatography measurement and T-RFLP (Terminal Restriction Fragment Length Polymorphism) revealed that the serial subculture of the enrichments shortened the time-course of the complete dechlorination of trichloroethene to ethene and altered the composition of bacterial communities. Complete dechlorination was observed in enrichments with significant abundance of *Dehalococcoides* sp. cultivated at 8 °C. Consortia incubated in fermenters at 18 °C accelerated the conversion of TCE to ethene by 7–14 days. Members of the enrichments belong to the phyla Bacteroidetes, Chloroflexi, Proteobacteria and Firmicutes. According to the operational taxonomic units, main differences between the composition of the enrichment incubated at 8 °C and 18 °C occurred with relative abundance of acetogenic and fermentative species. In addition to the temperature, the site-specific origin of the microbial communities and the solid phase applied during the fermentation technique contributed to the development of a unique microbial composition.

**Graphic abstract:**

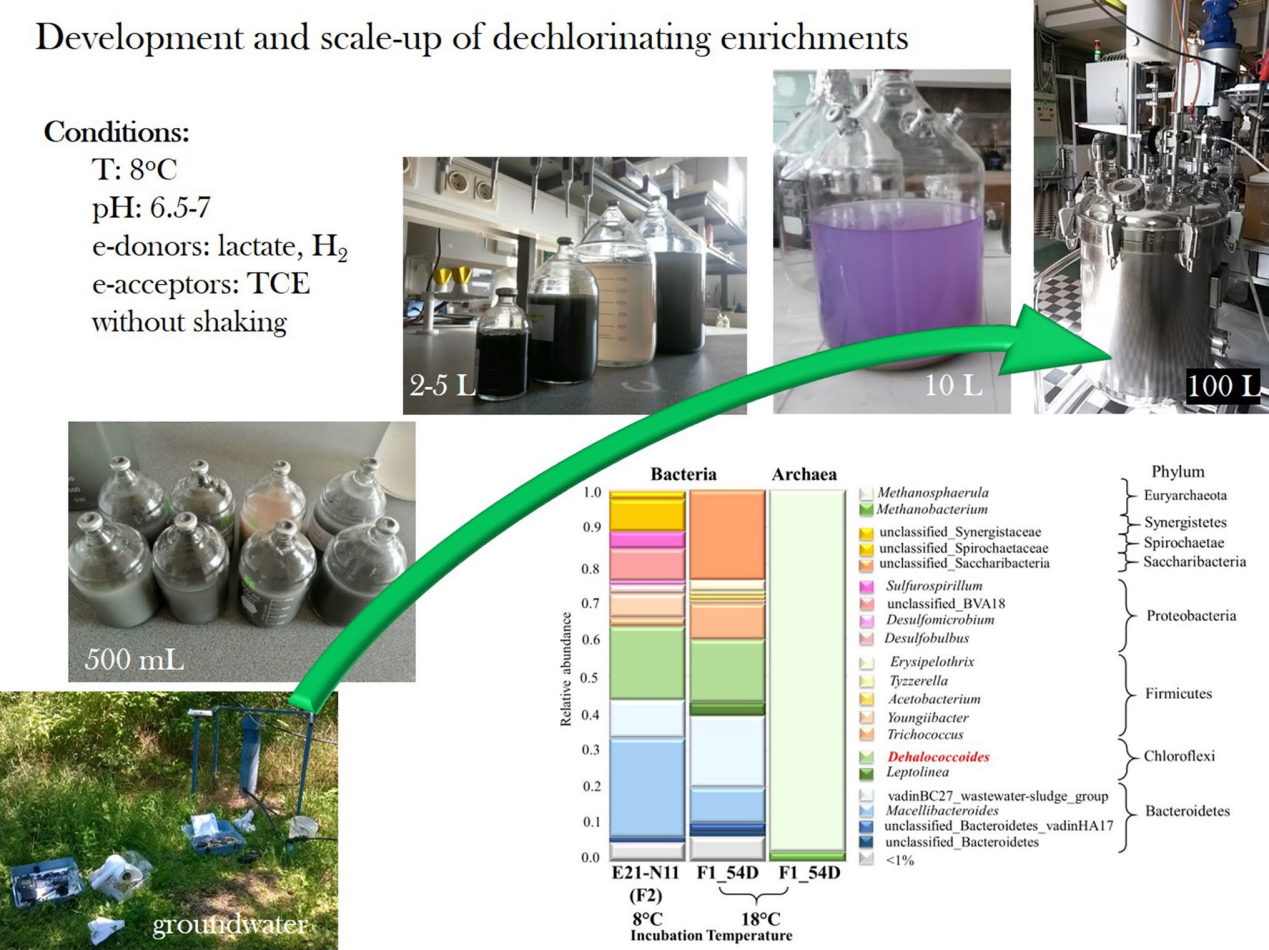

**Electronic supplementary material:**

The online version of this article (10.1007/s11274-020-2806-7) contains supplementary material, which is available to authorized users.

## Introduction

Short-chain halogenated aliphatic hydrocarbons (e.g.: tetrachloroethene—PCE, trichloroethene—TCE) are among the most toxic environmental pollutants of the twentieth century industry (Löffler et al. 2013b). These pollutants can accumulate in the environment causing contamination persisting for decades. TCE can be degraded under anaerobic conditions to non-toxic ethene via *cis*-dichloroethene (*cis-*DCE) and vinyl-chloride (VC) through the reductive dechlorination by organohalide-respiring microorganisms (McCarty 1997; Löffler et al. 2013b). Several bacteria are known to have the capability of reductive dechlorination, however, most of the isolated microorganisms can degrade PCE and TCE only to *cis*-DCE as *Desulfitobacterium* sp. (Gerritse et al. 1996), *Desulfomonile tiedjei* (Mohn and Tiedje 1990), *Dehalobacter restrictus* (Holliger et al. 1998), *Desulfuromonas chloroethenica* (Krumholz 1997), *Sulfurospirillum multivorans* (Luijten et al. 2003) and *Geobacter* sp. (Sung et al. 2006). Thus far, *Dehalococcoides* sp. had been identified as the only group of microorganisms capable of the complete dechlorination of chlorinated ethenes to non-toxic ethene, via the subsequent reduction of *cis*-DCE and VC (Maymó-Gatell et al. 1997; Adrian et al. 2007; Löffler et al. 2013a, b).

Reductive dechlorination can occur as natural attenuation or as a controlled approach such as bioaugmentation at sites contaminated with chlorinated hydrocarbons. In situ biological remediation is a feasible method to degrade short-chain halogenated hydrocarbons, however, microorganisms involved in dechlorination are usually underrepresented or absent in the contaminated areas (Löffler and Edwards 2006; Taş et al. 2010). Therefore, stimulating the autochthonous dechlorinating microbial population (biostimulation; i.e.: adding electron donors and/or nutrients) involved in the degradation or using dechlorinating inocula (bioaugmentation) containing *Dehalococcoides* sp. capable of complete dechlorination, provides a unique solution for remediation of contaminated sites. However, bioaugmentation process requires high cell density, upto 10^12^*Dehalococcoides* cells L^−1^ (Waller et al. 2005; Duhamel and Edwards 2006; Holmes et al. 2006; Hug et al. 2012; Delgado et al. 2014; Wen et al. 2017; Saiyari et al. 2018) in active cultures and the transformation of *cis*-DCE and VC, avoiding the accumulation of the nascent DCE and VC in the environment (Holmes et al. 2006). To establish dechlorinating enrichments, microbial communities were collected and enriched from different anaerobic sites contaminated with PCE and TCE (Duhamel et al. 2002; Holmes et al. 2006), and moreover from sediment of river and tropical mangrove and garden soils not exposed to contamination (He et al. 2005; Ziv-El et al. 2011; Delgado et al. 2014). The medium composition used for the enrichment and the maintenance of organohalide-respiring communities varied and were supplemented with different electron donors such as methanol and hydrogen in medium KB1 (Duhamel et al. 2002; Duhamel and Edwards 2006), lactate in medium ANAS (Holmes et al. 2006) and butyrate in medium DonnaII (Rowe et al. 2008). The developed cultures were maintained as two phases enrichments containing gas and liquid phases under different conditions in a final volume between 0.4 and 5.7 L, residence time between 16 days and 2 years, stirring and the temperature applied varied between 20 and 30 °C (Hug et al. 2012) coinciding the optimal temperature (25–40 °C) of *Dehalococcoides* sp. (Taş et al. 2010). Different strains of *Dehalococcoides* with unique reductive dehalogenase (*RDase*) genes were enriched (Duhamel et al. 2002; Waller et al. 2005; Duhamel and Edwards 2006; Holmes et al. 2006; Hug et al. 2012) according to the composition of the active cultures.

Bioaugmentation agents apply allochthonous microbial consortia for bioremediation of different sites contaminated with chlorinated aliphatic hydrocarbons. Our aim was to develop a bioremediation technique with the application of site specific autochthonous bioaugmentation agent. Microbial communities enriched from contaminated sites can be applied as site specific bioaugmentation cultures providing an effective strategy to enhance bioremediation processes. The application of predominately autochthonous microbial community adapted to certain pollution and environmental conditions has a greater chance of being effective. The main goal of our work was to produce a site specific bioaugmentation culture using enrichments established in microcosm experiments, and to develop a fast and reliable enrichment method to achieve appropriate inoculum quantity. For this purpose, dechlorinating microbial consortia were collected from PCE and TCE contaminated groundwater. The collected microbial communities were enriched by anaerobic microcosm experiments containing clay mineral as solid phase. Optimization of the transfer process of the anaerobic microcosms at bench- (500 mL, 2 L, 5 L and 10 L) and industrial-scale (in 100 L working volume fermenter) was the main target of our experiments. Considering the incubation temperature (20 °C to 30 °C) of the developed dechlorinating microbial cultures, which exceeded the temperature of the groundwater that is below 20 °C. Enrichment of organohalide-respiring microbial communities need to be adapted to temperature comparable to groundwater.

In our work, we focused on the following points of scientific interest. We intended to explore in depth the microbial communities including the composition of *Dehalococcoides* sp. strains and varieties among the reductive dehalogenase genes playing a role in dechlorination process. Next, the effects of the inoculation and serial transfers upto 100 L were investigated: the composition of the microbial community and the diversity of *Dehalococcoides* sp. and thereby the diversity of the key dehalogenase genes impacting the progress of dechlorination activities were monitored throughout the process. Finally, we examined the correlation between the serial transfers and the changes in the composition of the organohalide-respiring bacterial communities and its effect on the dechlorination process. Polyphasic approach was applied to answer these questions. The effectiveness of the dechlorination was monitored based on the concentration change of the intermediate products of the TCE dechlorination by gas chromatography measurement. The bacterial community composition of the groundwater and its changes in enrichments were assessed by Terminal Restriction Fragment Length Polymorphism (T-RFLP) analysis based on 16S rRNA gene over the time. To reveal the composition of the *Dehalococcoides* sp. strains, 16S rRNA gene and three *RDase* genes such as trichloroethene (*tceA*) and vinyl-chloride reductase (*vcrA, bvcA*) genes were sequenced. Next generation sequencing was applied to get a comprehensive insight into the microbial community of enrichments established in 100 L volume.

## Materials and methods

### Samples

**Fig. 1:**
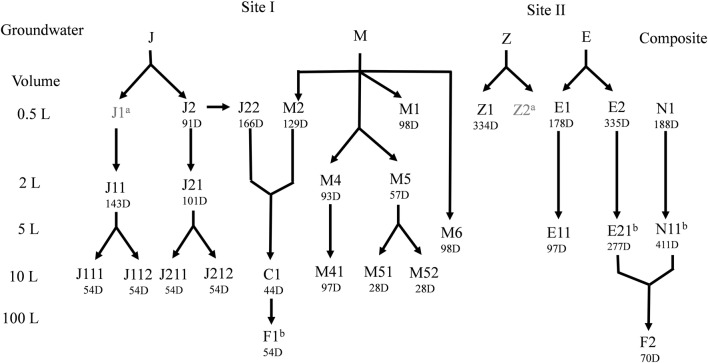
Sample nomenclature and the scheme of serial transfer procedure of the enrichments. Sampling days are indicated beneath the sample names. Arrows indicate the transfer direction of the enrichments. ^a^No sampling for microbiological analysis; ^b^samples used for next generation sequencing

Groundwater samples were collected from two sites (I and II) contaminated with halogenated aliphatic hydrocarbons. The main contaminants were PCE and TCE leaked from storage tanks. Biostimulation was applied beforehand at site I, involving injection of organic substrates into wells. Considering site II no remediation process was carried out previously. Groundwater samples were collected from two wells at each site, sample names refer as follows: J and M samples collected from site I; Z and E samples collected from site II (Fig. 1). Groundwater samples were collected using low-flow technique in anaerobically sterilized 2.0 L and 0.5 L bottles filled up without headspace for microbiological and chemical analysis, respectively. Water samples were kept at 10 °C during transfer to the laboratory and were processed immediately upon arrival. On site measurement of dissolved oxygen concentration (DO), pH, temperature (T), specific electrical conductivity (EC) and oxidation–reduction potential (ORP) was made using a multimeter (WTW Type Multi 350i, Weilheim, Germany). Water chemistry analysis were performed by the accredited laboratory of Wessling Hungary Ltd (Hungary, Budapest).

### Microcosms and enrichment preparation

Groundwater samples J, M, Z and E were used as inocula to set up enrichments using microcosm experiments. One microcosm group was inoculated with the composite of microbial consortia derived from J, E and Z groundwater samples in ratio 1:1:1 which is referred as N. Five groups of enrichments were established in minimum two parallel (Fig. 1). The enrichments were amended with hydrogen and sour whey containing acetate and lactate as carbon and energy sources to enrich *Dehalococcoides* sp. and enhance reductive dechlorination. The enrichments prepared in microcosms with 500 mL volume were transferred into same size bottle (500 mL) and/or into 2 L, 5 L and 10 L mesocosms and finally to fermenters with 100 L volume according to the enrichment scheme shown in Fig. 1. Microcosms were prepared as described by Mészáros et al. (2013). Briefly, the autochthonous microbial community of 2.0 L groundwater samples were concentrated on a 0.22 μm pore size cellulose nitrate filter (Millipore, Billerica, USA) under CO_2_ atmosphere to avoid oxygen dissolution during vacuum filtration. The membranes containing the autochthonous microbial communities were shaken in 40 mL sterile anaerobic medium (Zinder 1998) overnight to produce inocula. Enrichments of different volumes were prepared with the ratio of gas, liquid and solid phases as follows: headspace ratio 1:3; solid to liquid ratio 1:17. The liquid phase of the microrcosms was 425 mL anaerobic medium (Zinder 1998) purged with 5.0 nitrogen gas (grade 99.99%) (Linde, Munich, Germany) to maintain anaerobic conditions and 23 g clay mineral (particle size: < 1.6 mm) was used as solid phase. Resazurine (1 g L^−1^) was added to the enrichments as an indicator of the redox condition. Bottles were sterilized for 50 min (121 °C, 1 atm) and the headspace was changed to nitrogen gas. After sterilization NaHCO_3_ (1 g L^−1^), Na_2_S (25 mg L^−1^), vitamins (Zinder 1998), sodium-acetate (2 mM) (Sigma-Aldrich) as carbon and energy source and sour whey (total organic carbon content: 73 g L^−1^) containing lactate as carbon source and supplement nutrient were added to the enrichments. The pH was adjusted to 6.5–7.0 with acetic-acid (5%). TCE (415 μmol L^−1^) (Sigma-Aldrich, spectrophotometric grade ≥ 99.5%) was added as electron acceptor and it was re-dosed after exhausted from the headspace. Furthermore, upon each transfer of enrichments additional TCE was supplied. As last step, enrichments (500 mL) were inoculated with seed cultures originated from different groundwater samples. The volume of the added inoculum was 10% v/v of the liquid phase in each transfer of enrichments. Abiotic control microcosms were set up without any carbon source, electron donors and inocula and were sterilized on three consecutive days. Enrichments were incubated upside down in dark without shaking at 8 °C. Hydrogen gas (grade: 80% H_2_ and 20% CO_2_) (Linde, Munich, Germany) as electron donor (overpressure) was added monthly. Anaerobic fermenter was designed for cultivation of dechlorinating enrichments in 100 L volume. Fermentation protocol was elaborated for anaerobic inoculation, sampling, feeding and dosage for long term storage. Test run was started to investigate all the required conditions and features throughout the cultivation in the 100 L prototype fermenter.

### The seed train scheme of the enrichments from bench-scale to industrial-scale

Three different seed trains schemes were applied including two or three transfers in order to achieve appropriate inoculum quantity for the inoculation of anaerobic fermenters with a final volume of 100 L (Fig. 1). Considering the 10% v/v volume of the inoculum, enrichments inoculated with groundwater M were established in 2 L and 5 L volume, as well (Fig. 1). Enrichments prepared in microcosms with 500 mL volume and inoculated with the samples of groundwater J and E were transferred to 2 L and 5 L, respectively (Fig. 1). Thereafter enrichments prepared in 2 L or 5 L volume were used to inoculate modified Duran laboratory bottles of 10 L volume or directly to the anaerobic fermenter. Two anaerobic fermenters were inoculated with composite enrichments applying transfer processes as shown in Fig. 1. Two anaerobic fermenters (F1 and F2) were inoculated with the enrichment C1 prepared in 10 L volume and the composite of enrichments E21 and N11 prepared in 5–5 L volume (Fig. 1). Enrichments were used as seed culture after TCE had been dechlorinated into *cis*-DCE and to ethene.

### Analytical methods

The dynamic characteristic of the dechlorination sequence were monitored by using a HP5890 gas chromatograph (Agilent Technologies Inc., Santa Clara, CA, USA) with flame ionization detection equipped with HP-PLOT Q type column (15 m × 0.53 mm). The method is suitable to separate methane, ethene, vinyl-chloride, *cis*- and *trans*-DCE, TCE and PCE providing information on the dominant daughter products of TCE dechlorination appearing in the headspace of the enrichments. The carrier gas was Grade 5.0 helium (Linde, Munich, Germany). The injector was operated in split mode at a ratio 1:20. The temperature protocol was as follows: 60 °C 2 min, 25 °C min^−1^ to 250 °C, the temperature of the detector and the injector was 250 °C. The calibration standards were prepared with same phase ratios and volumes as the microcosms and were stored and sampled in the same way. The injected volume of the headspace was 100 μL during manual injection using gas-tight Hamilton (Reno, USA) syringe. Before gas-chromatography measurements enrichments were incubated for 24 h at 20 °C to develop the equilibrium of the volatile compounds among the three phases.

Total organic carbon content (TOC) was measured applying a Multi N/C 2100S TC/TN analyser (Analytik Jena, Germany).

### Nucleic acid extraction

For the extraction of the community DNA 2.0 L of contaminated groundwater was filtered through 0.22 μm pore size cellulose nitrate filter (Millipore, Billerica, USA). Before the transferring procedure 2 mL enrichment samples were collected using a sterile syringe purged with nitrogen gas. Enrichment samples were centrifuged at 12,000×*g* for 5 min. DNA was extracted from membrane filters and from the pellets derived from groundwater and enrichments, respectively, using Ultraclean™ Soil DNA Isolation Kit (MoBio Laboratories Inc. Carlsbad, USA) according to manufacturer’s instructions, with a modification at the physical cell disruption step. The samples were shaken in “Bead Solution tubes” (MoBio Laboratories) containing microbeads at 25 Hz for 2 min using mixer mill MM301 (Retsch, Haan, Germany).

### Taxon-specific 16S rRNA and reductive dehalogenase (*RDase*) genes targeted PCR

Taxon-specific 16S rRNA gene was used to detect the presence of organohalide-respiring microorganisms, such as *Dehalococcoides* sp., *Desulfitobacterium dehalogenans*, *Dehalobacter restrictus*, *Desulfomonile tiedjei, Desulfuromonas chloroethenica* and *Geobacter* sp. *Dehalococcoides* sp. strains related *RDase* genes such as trichloroethene reductase (*tceA*) and vinyl-chloride reductases (*vcrA* and *bvcA*) also amplified by PCR. The applied primer sets are presented in Table S1. *Dehalococcoides* sp. specific 16S rRNA gene sequences were amplified in nested PCR approach as described by Révész et al. (2006). Thermal profiles of the PCR reactions were as follows: initial denaturation at 98 °C for 5 min, followed by 32 amplification cycles of 30 s at 94 °C, for 45 s at target sequence-specific annealing temperature as follows: *Dehalococcoides* sp. and *vcrA* at 55 °C, *D. restrictus* and *D. dehalogenans* at 65 °C, *D. chloroethenica* and *D. tiedjei* at 64 °C, *Geobacter* sp., *tceA* and *bvcA* at 59 °C, 49 °C and 52 °C, respectively, 1 min at 72 °C, followed by final extension at 72 °C for 10 min. The PCR reaction mixture contained 1 U of LC*Taq* DNA Polymerase (Thermo Fisher Scientific Inc., Waltham, MA, USA), 200 μM of each deoxynucleoside triphosphate, 1 × *Taq* buffer with (NH_4_)_2_SO_4_ (Thermo Fisher Scientific Inc. USA), 2 mM MgCl_2_, 0.32 μM of each primer, and 1–2 μL DNA template in final volume of 50 μL.

### T-RFLP analysis

The bacterial community composition and their changes in enrichments and in fermenters were assessed by T-RFLP analysis over the time. Bacteria 16S rRNA gene was amplified using 27F forward primer labelled with 5′-hexachloro-fluorescein and 519R reverse primer (Lane 1991). T-RFLP analysis was performed as described by Nagymáté et al. (2016) with the differences that *Alu*I and *Bsh*1236I (Thermo Fisher Scientific Inc., USA) endonucleases applied. Electropherograms were analysed with the GeneMapper software (Applied Biosystems; Version 3.7). Fragment size longer than 50 base pairs and fluorescence intensity higher than 50 fluorescence units (peak height) were used to define T-RF peaks. Data matrices were created with T-RF’s ≥ 1% relative abundance using T-RFLP Analysis Expedited (T-REX) software (Culman et al. 2009). Statistical data analysis was performed by Principal Component Analysis (PCA) using PAST software system (Hammer et al. 2001). To verify the significance of the separation among the microbial community of the different enrichment groups considering the used inoculum and the transferring steps, permutational multivariate analysis of variance (PERMANOVA, 999 permutations) was applied using PAST software package.

### Sequence analyses

The 16S rRNA gene fragment of genus *Dehalococcoides* and the three *RDase* genes (*tceA*, *vcrA* and *bvcA*) were sequenced by Sanger method using group specific forward primers at LGC Ltd. (Berlin, Germany). Sequences were aligned by Basic Local Alignment Search Tool (https://www.ncbi.nih.gov/BLAST) using the GenBank nucleotide database. Operational taxonomic units (OTUs) were assigned at 95% and 97% similarity threshold levels, representing microbial genera and species, respectively, according to Tindall et al. (2010). Sequences obtained in this study were submitted to European Nucleotide Archive (Accession numbers: LR026649-LR026661 for 16S rRNA gene and LR031310-LR031336 for reductive dehalogenase genes).

Prior to inoculation of the fermenter F2 composite sample was prepared by mixing enrichments E21 and N11. The microbial communities of the fermenter F1 and the composite enrichment after 54 and 70 days incubation, respectively, were assessed by high throughput pyrosequencing of the V1-V3 region of 16S rRNA gene. Bacteria 16S rRNA gene was amplified using the same universal primer set as for T-RFLP analyses (Nagymáté et al. 2016). Archaea 16S rRNA gene was amplified using CS1-A519F and CS2-A-855R (Klindworth et al. 2013) primer sets with the following thermal conditions: initial denaturation at 98 °C for 5 min, followed by 25 amplification cycles of 30 s at 95 °C, for 30 s at 60 °C, 30 s at 72 °C, followed by final extension at 72 °C for 10 min. The forward primers were fused with sequencing barcodes and adapters. The amplification and subsequent next generation sequencing of Bacteria and Archaea 16S rRNA genes was performed as described by Krett et al. (2017). Emulsion PCR, amplicon library processing and pyrosequencing were performed on GS Junior sequencing platform according to the Lib-L protocol of the manufacturer (Roche/454 Life Sciences). Bioinformatics analyses of the resulting sequence reads were carried out with mothur (Schloss et al. 2009) as described in detail by Szabó et al. (2017). Sequences were aligned and classified by the SINA v1.2.11 aligner tool (Pruesse et al. 2012) using the ARB-SILVA SSU NR reference database—SILVA Release 123 (Quast et al. 2013). Sequences were submitted to the NCBI SRA database and available under the BioSample accession number SAMN10024392.

Representatives of the established operational taxonomic units (OTUs) were selected for in silico determination of the individual T-RF peaks of the community members especially the *Dehalococcoides* sp. For this purpose, unique T-RF’s of the OTUs were identified by MEGA6 program based on the recognition site of restriction endonucleases applied for T-RFLP analysis (*Alu*I and *Bsh*1236I) (Tamura et al. 2013).

## Results

### Physico-chemical and molecular biological data of the two contaminated sites

Physico-chemical parameters of the groundwater samples are listed in Table 1. The groundwater samples, originated from geographically-distinct sites, were characterized with neutral pH 6.8–7.4 and comparable temperature ranging 13.1–14.5 °C. According to the specific electrical conductivity measurements the groundwater samples showed remarkable differences. Sample from well J was characterized with the highest value 2260 µS cm^−1^ caused by the injection of organic substrates, while the specific electrical conductivity of the other samples was lower ranged in 1031–1260 µS cm^−1^ (Table 1). The DO concentration was low varying between 0.11 and 1.27 mg L^−1^ and the oxidation–reduction potential was below − 94 mV (Table 1) at site I indicating prevailing anoxic conditions. Concentration of ammonium- and phosphate-ions were slightly higher in well E, while TOC and nitrate concentrations were higher in well J and M (Table 1). Chemical analyses of groundwater revealed high volatile chlorinated hydrocarbons (VOCl) contamination (Table 1) exceeding 65 mg L^−1^ concentration with main compounds of *cis*-DCE, VC and traces of TCE at site I. Moreover, methane (3.3–14.2 mg L^−1^), ethene (0.97–4.3 mg L^−1^) and high chloride-ion concentration (72–266 mg L^−1^) indicated ongoing reductive dechlorination at site I (Table 1). Significantly lower VOCl contamination was revealed at site II with TCE and *cis*-DCE as main compounds and traces of PCE, *trans*-DCE and VC (Table 1). Though, ethene concentration was not detected in the wells of the site II indicating partial dechlorination process under natural conditions.

**Table 1:** Physico-chemical characteristics of groundwater samples collected from the wells of the contaminated sites; n.d. no data available due to technical failure

	Site I		Site II	
	J	M	Z	E
pH	7.48	7.47	6.83	8.15
T (°C)	14.3	14.5	13.3	14.1
DO (mg L^−1^)	0.5	0.3	0.1	1.3
ORP (mV)	− 94	-458	n.d	n.d
EC (μS cm^−1^)	2260	1260	1022	1031
NH_4_^+^-N (mg L^−1^)	1.3	0.6	0.5	2.9
NO_2_^−^-N (mg L^−1^)	< 0.01	< 0.01	< 0.01	< 0.01
NO_3_^−^-N (mg L^−1^)	1.5	1.1	0.5	0.8
Cl^−^ (mg L^−1^)	266	72	39	43
Fe (mg L^−1^)	8.20	1.36	9.07	8.08
SO_4_^2−^ (mg L^−1^)	6	100	129	74
PO_4_^3−^ (mg L^−1^)	0.66	< 0.5	1.68	15.1
TOC (mg L^−1^)	363	400	23	26
PCE (μg L^−1^)	< 1	< 1	< 1	15.6
TCE (μg L^−1^)	25	47	45	246
*cis*-DCE (μg L^−1^)	58,900	1350	82	187
*trans*-DCE (μg L^−1^)	< 1	30	< 1	1
VC (μg L^−1^)	66,600	390	1	6
ethene (μg L^−1^)	970	4300	< 5000	< 5000
methane (μg L^−1^)	3310	14,290	< 5000	< 5000
VOCl (μg L^−1^)	65,373	1437	128	450

The volatile chlorinated hydrocarbons concentration abbreviated as VOCl

Regarding the targeted detection of microorganisms capable of reductive dechlorination only minor differences were observed among the samples with genera *Geobacter*, *Dehalobacter, Desulfitobacterium, Desulfomonile* and *Desulfuromonas* capable of partially dechlorinated PCE and TCE to *cis-*DCE (DeWeerd et al. 1990; Utkin et al. 1994; Krumholz 1997). 16S rRNA gene amplicons of *Geobacter* were detected in all examined groundwater. The sequence analysis of 16S rRNA gene revealed the presence of *Geobacter lovleyi* strain SZ in the groundwater. Members of genus *Dehalococcoides* were present in all examined groundwater. However, the distribution of *Dehalococcoides* sp. related *RDase* genes such as *tceA*, *vcrA* and *bvcA* genes showed differences (Table 2). Amplicons of the three *RDase* genes were detected in both wells of site I (J and M) and in groundwater Z at site II. Amplicon of *tceA* gene was not detected in groundwater E. The sequence analysis of 16S rRNA gene fragment of *Dehalococcoides* sp. obtained from groundwater revealed 100% sequence similarity to *Dehalococcoides* sp. GT, FL2, CBDB1, BAV1 and BTF08 strains like microorganisms. Sequence of *tceA* and *vcrA* genes retrieved from sample J revealed the presence of *Dehalococcoides* sp. BTF08 (Pöritz et al. 2013) and *Dehalococcoides* sp. UCH007 strains like microorganisms (Uchino et al. 2015), respectively (Table 2). Sequence analysis of *vcrA* gene obtained from groundwater E and M resulted *Dehalococcoides* sp. UCH007 and *Dehalococcoides* sp. BTF08 strains like microorganisms, respectively (Table 2). However, *tceA* amplicon retrieved from samples M and Z and *vcrA* gene obtained from groundwater Z resulted ambiguous mixed base sequences. The sequence analyses of the *bvcA* gene confirmed the presence of *Dehalococcoides* sp. BAV1 strain like microorganism (Table 2) in each groundwater. According to the sequence analyses of the three *RDase* genes diverse *Dehalococcoides* population was present in the examined sites.

**Table 2:** The table shows the main decomposition intermediates of TCE and the closest *Dehalococcoides* sp. relatives based on the reductive dehalogenase gene sequences

Sample name	Volume of the enrichment (L)	Incubation period (days)	Dechlorination intermediates	*Dehalococcoides mccartyi*	Reductive dehalogenase genes with assigned function		
				relative abundance (%)	*tceA*	*vcrA*	*bvcA*
Z			TCE, *cis*-DCE	< 1	impure	impure	*Dehalococcoides* sp. BAV1 CP000688 (99%)
Z1	0.5	334	*cis*-DCE	< 1	X	X	n.d
E			TCE, *cis*-DCE	< 1	n.d	*Dehalococcoides* sp. UCH007 AP014722 (100%)	*Dehalococcoides* sp. BAV1 CP000688 (99%)
E1	0.5	178	*cis*-DCE	< 1	n.d	impure	impure
E11	5	97	TCE, *cis*-DCE	< 1	n.d	X	n.d
E2	0.5	335	*cis*-DCE	< 1	n.d	X	n.d
E21	5	277	*cis*-DCE	< 1	X	n.d	X
J			*cis-*DCE	27.9	*Dehalococcoides* sp. BTF08 CP004080 (100%)	*Dehalococcoides* sp. UCH007 AP014722 (100%) *Dehalococcoides* sp. ANAS2 HM241732 (100%)	*Dehalococcoides* sp. BAV1 CP000688 (99%)
J2	0.5	91	*cis-*DCE	1.3	X	X	X
J22	0.5	166	*trans-*DCE, Ethene	7.2	X	X	n.d
J21	2	101	VC, Ethene	19	*Dehalococcoides* sp. BTF08 CP004080 (99%)	impure	*Dehalococcoides* sp. BAV1 CP000688 (98%)
J211	10	54	VC, Ethene	8.9	impure	n.d	*Dehalococcoides* sp. BAV1 CP000688 (98%)
J212	10	54	VC, Ethene	6.0	*Dehalococcoides* sp. BTF08 CP004080 (99%)	n.d	*Dehalococcoides* sp. BAV1 CP000688 (98%)
J11	2	143	*cis*-DCE	< 1	X	X	X
J111	10	54	TCE, *cis*-DCE	< 1	n.d	n.d	n.d
J112	10	54	*cis*-DCE	< 1	n.d	X	X
M			*cis*-DCE	< 1	impure	*Dehalococcoides* sp. BTF08 CP004080 (100%) *Dehalococcoides* sp. GT CP001924 (100%)	*Dehalococcoides* sp. BAV1 CP000688 (98%)
M1	0.5	98	*cis*-DCE*,* VC, Ethene	< 1	n.d	X	X
M2	0.5	129	Ethene	17.6	n.d	X	X
M4	2	93	*cis*-DCE*,* VC, Ethene	< 1	n.d	X	X
M41	10	97	VC, Ethene	2.7	n.d	*Dehalococcoides sp.* BTF08 CP004080 (100%) *Dehalococcoides sp.* GT CP001924 (100%)	*Dehalococcoides* sp. BAV1 CP000688 (98%)
M5	2	57	*cis*-DCE	< 1	n.d	*Dehalococcoides* sp. BTF08 CP004080 (100%) *Dehalococcoides* sp. GT CP001924 (100%)	n.d
M51	10	28	*cis*-DCE	< 1	n.d	*Dehalococcoides* sp. BTF08 CP004080 (100%) *Dehalococcoides* sp. GT CP001924 (100%)	n.d
M52	10	28	*cis*-DCE	< 1	n.d	*Dehalococcoides* sp. BTF08 CP004080 (100%) *Dehalococcoides* sp. GT CP001924 (100%)	n.d
M6	5	98	*cis*-DCE*,* VC	< 1	n.d	X	X
N1	0.5	188	*cis*-DCE	2.5	*Dehalococcoides* sp. BTF08 CP004080 (99%) *Dehalococcoides* sp. UCH007 AP014722 (99%)	n.d	*Dehalococcoides* sp. BAV1 CP000688 (98%)
N11	5	411	VC	29.3	*Dehalococcoides* sp. BTF08 CP004080 (99%) *Dehalococcoides* sp. AP017649 (99%)	n.d	*Dehalococcoides* sp. BAV1 CP000688 (98%)
C1	10	44	TCE, Ethene	10.5	Impure	*Dehalococcoides* sp. BTF08 CP004080 (100%) *Dehalococcoides* sp. GT CP001924 (100%)	*Dehalococcoides* sp. BAV1 CP000688 (98%)
F1	100	54	*trans-*DCE, Ethene	15	X	*Dehalococcoides* sp. BTF08 CP004080 (100%) *Dehalococcoides* sp. GT CP001924 (100%)	*Dehalococcoides* sp. BAV1 CP000688 (98%)
F2	100	70	VC, Ethene	15.7	X	*Dehalococcoides* sp. BTF08 CP004080 (100%) *Dehalococcoides* sp. GT CP001924 (100%)	impure

The detection of catabolic reductive dehalogenase genes and the *Dehalococcoides* sp. (abbreviated as DHC) with its ratio in the bacterial community based on in silico data and the sampling days are indicated. X indicates the presence of the reductive dehalogenase genes without phylogenetic information. Due to the low yield of PCR reactions the sequence analyses of the *tceA* gene derived from F1 and F2 samples could not be performed; n.d: not detected by PCR

### TCE dechlorination in enrichments

The enrichments inoculated with microbial community derived from groundwater with different origin had different lag times prior to dechlorination and were characterized with different TCE degradation potential. Dechlorination of TCE with the accumulation of *cis*-DCE started 15 to 57 days following the establishment of the enrichments in 500 mL volume.

**Fig. 2:**
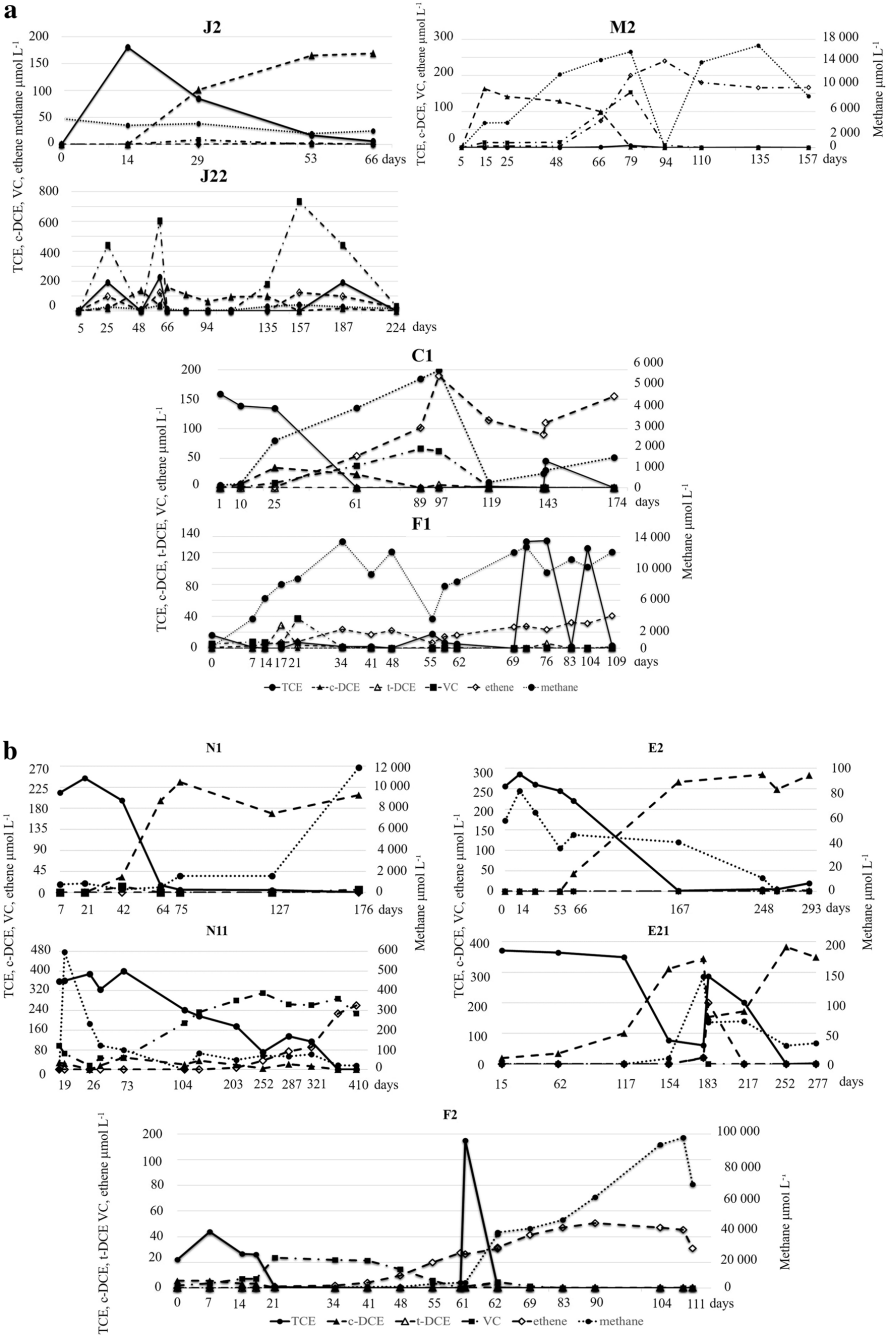
TCE transformation to daughter products in the enrichments transferred to industrial scale by two different transfer schemes including two or three transfer steps to achieve bioaugmentation inoculum over time. Inoculation pathways of fermenters F1 (**a**) and F2 (**b**)

The enrichments of groups J and M maintained in different volume were characterized with the most efficient TCE degradation potential, since TCE was transformed to *cis*-DCE in less than 30 days (Fig. 2). Partial dechlorination with the accumulation of vinyl-chloride was observed in the transferred (second stage) enrichments (500 mL and 2 L; J11, J21 and J22) (Table 2; Fig. 2) showing increased TCE degradation efficiency compared to first stage enrichments prepared in 500 mL. Enrichments J11 and J21 were transferred to 10 L volume scale (Fig. 1, Table 2) after short-term (101 days—accumulation of *cis*-DCE) and long-term (493 days—accumulation of ethene) incubation, respectively. Complete dechlorination of TCE, with increasing concentration of *cis*-DCE, VC and accumulation of ethene, was achieved in the 10 L volume enrichments by 126 to 291 days. Considering the time-scale of the three-step transferring process from groundwater to 10 L, complete dechlorination of TCE to ethene was observed in enrichments assigned as J by 392 or 619 days applying short- and long-term incubation, respectively.

Composite enrichments were applied to inoculate fermenters F1 and F2 to shorten the time-scale of dechlorination. Two composite enrichments, C1 with 10 L volume and the composite of enrichments N11, E21 with 5–5 L volume, were used to inoculate the fermenters F1 and F2, respectively (Figs. 1, 2). Enrichment C1 was characterized with accelerated dechlorination since all TCE was transformed in line with increasing ethene concentration by day 61 (Fig. 2). Accelerated dechlorination was observed in the composite enrichment N11 with the accumulation of VC and ethene by day 19 and 252, respectively. However, in the enrichment E21 inoculated with groundwater originated from non-treated site II, TCE was dechlorinated mainly to *cis*-DCE with the detection of traces of VC and ethene during the 277 days incubation period. Our results imply fast TCE degradation to *cis*-DCE and VC, whereas the further dechlorination step with the presence of ethene was slower suggesting co-metabolic VC dechlorination in large scale enrichments. Considering the intermediates of the dechlorination of TCE, differences were observed between the two fermenters. TCE was transformed to ethene with the periodic detection of *trans*-DCE in F1 in 14 days. While, in the fermenter F2 low amounts of *cis*-DCE and VC were detected during the TCE conversion to ethene taking only 7 days. Moreover, the parallel headspace measurements showed significant amount of methane (Fig. 2) providing indirect evidence for the presence of methanogenic Archaea.

Conforming the analytical results enrichments maintained capabilities to dechlorinate TCE after the transferring procedures, implying the effectiveness of the applied anaerobic inoculation and fermentation processes. However, based on the gas chromatography measurements, accumulation of the daughter products of the TCE dechlorination was observed, exceeding the intial molar concentration of TCE. Presumably, TCE was adsorbed to the applied solid phase causing lower concentration in the headspace. This phenomenon was further strengthened by periodic addition of TCE causing more extent accumulation of volatile products such as ethene and VC and to a lesser extent *cis*-DCE in the headspace.

### The bacterial community structure in the groundwater and in enrichments

**Fig. 3:**
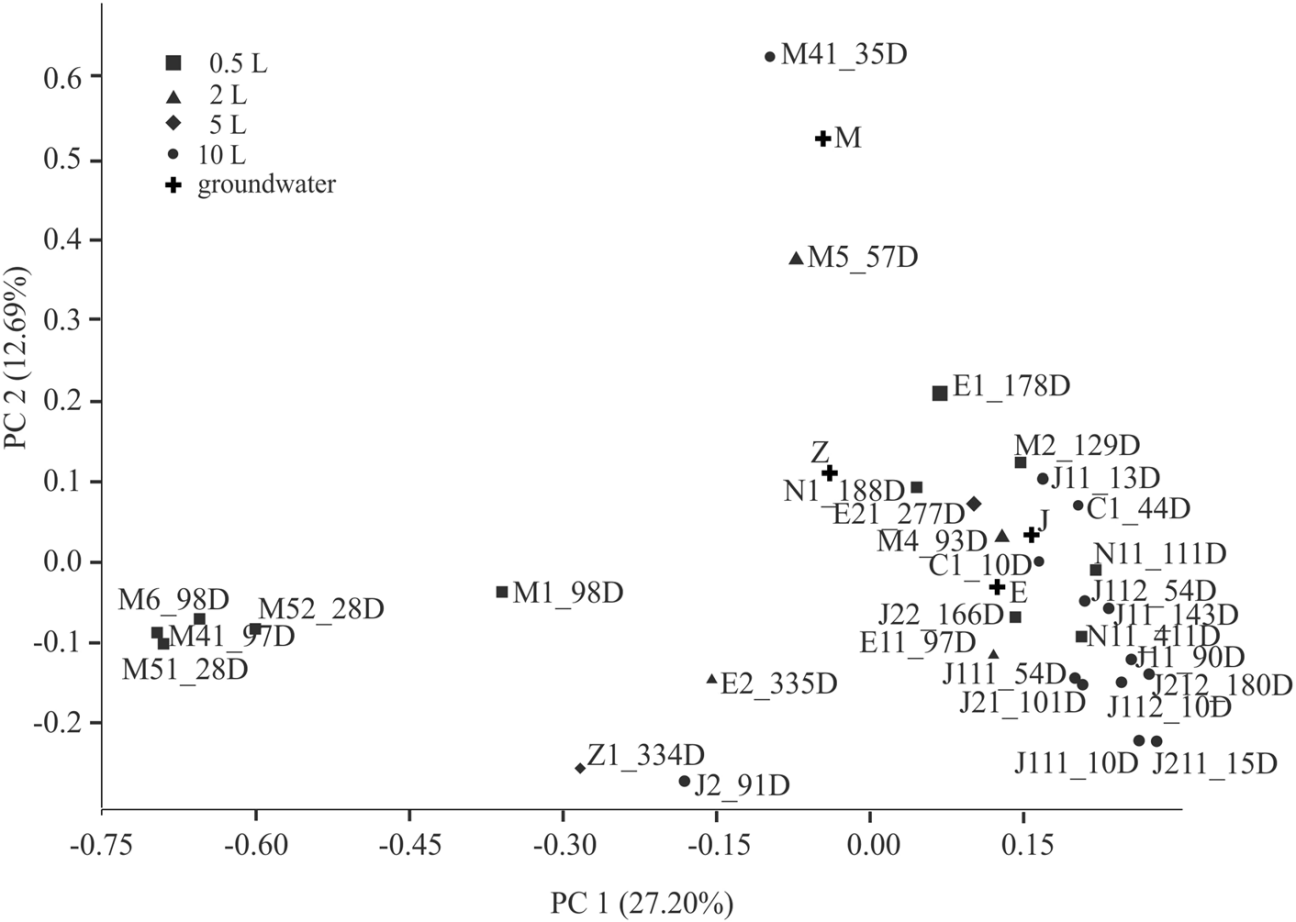
Two-dimensional principal component analysis (PCA) plot of T-RFLP data retrieved from bacterial communities of groundwater and enrichments. Sampling days are indicated following the sample names (e.g.: *M5_57D*)

To assess the changes in the bacterial communities 16S T-RFLP analyses were applied throughout the experiment. The bacterial community of the collected groundwater samples characterized with different physico-chemical parameters showed remarkable separation according to the PCA ordination of 16S T-RFLP fingerprints based on the first two principal components (Fig. 3) suggesting differently structured communities. Based on the first two principal components the enrichments inoculated with microbial communities derived from distinct groundwater samples also showed significant differences (F_PERMANOVA_: 2.58; p: 0.0001), furthermore the microbial community of duplicate enrichments showed only slight separation (Fig. 3). Our results imply that the selective enrichment procedure caused significant (F_PERMANOVA_: 2.58; p: 0.0001) changes in the bacterial community composition of enrichments compared to groundwater (Fig. 3). The bacterial community structures were significantly influenced by transferring steps also, especially the third transfer step (F_PERMANOVA_: 1.76; p: 0.013), presumably related to the length of incubation. Furthermore, according to the presence of *cis*-DCE and ethene as dominant by-products of TCE dechlorination, significant (F_PERMANOVA_: 1.65; p: 0.018) differences were observed in the community structures suggesting the alteration and the slight decrease in the diversity indices (data not shown) of the bacterial community with the progress of dechlorination process regardless of the volume of the enrichments.

### Taxon-specific detection and analysis of reductive dehalogenase genes

Presence of bacteria capable of reductive dechlorination (such as members of genera *Geobacter*, *Desulfitobacterium, Desulfomonile, Desulfuromonas* and *Dehalococcoides*) were assayed in the enrichments after TCE was transformed. The 16S rRNA gene amplicons of *Geobacter* spp., identical to *Geobacter lovleyi* strain SZ (100% sequence similarity), were detected in the enrichments similarly to the groundwater. In all enrichments characterized with partial and complete dechlorination *Dehalococcoides*-like sequences could be detected by direct and nested PCR approach. Sequence analysis of 16S rRNA gene, revealed two ribotypes of *Dehalococcoides* in the enrichments. The retrieved sequences were identical (100% sequence similarity) to *Dehalococcoides* sp. GT, FL2, CBDB1, BAV1 and BTF08 strains like microorganisms as was observed in the case of the groundwater samples. Another ribotype of *Dehalococcoides* was obtained from enrichment inoculated with groundwater M showing 100% similarity to *Dehalococcoides mccartyi* 195 (T).

Contrary, the detection of *Dehalococcoides* related *RDase* genes (*tceA, vcrA* and *bvcA*) showed differences (Table 2). Amplicons of *tceA* gene were detected in enrichment groups J and N predominantly (Table 2). Considering the presence of the *tceA* gene differences were observed in the case of enrichments M and E. Groundwater M was characterized with the presence of *tceA* gene, which was not detected in the enrichments. In enrichments referred as E the presence of *tceA* gene was only detected after selective enrichment steps, suggesting low copy number of *tceA* gene in groundwater E. Both vinyl-chloride reductase genes (*vcrA* and *bvcA*) were detected in enrichments (500 mL) inoculated with groundwater (Table 2). However, enrichments transferred to 5 L and 10 L volumes were usually characterized with the presence of only one vinyl-chloride reductase gene (Table 2). Amplicons of all the three *RDase* genes were merely detected in two enrichments inoculated with groundwater J and in the two fermenters suggesting the presence of a non-detectable minor *Dehalococcoides* sp. population in enrichments. The phylogenetic analyses of *tceA*, *vcrA* and *bvcA* genes revealed the presence of *Dehalococcoides* sp. BTF08, UHC007, GT and BAV1 strains like microorganisms (Table 2).

### NGS results

**Fig. 4:**
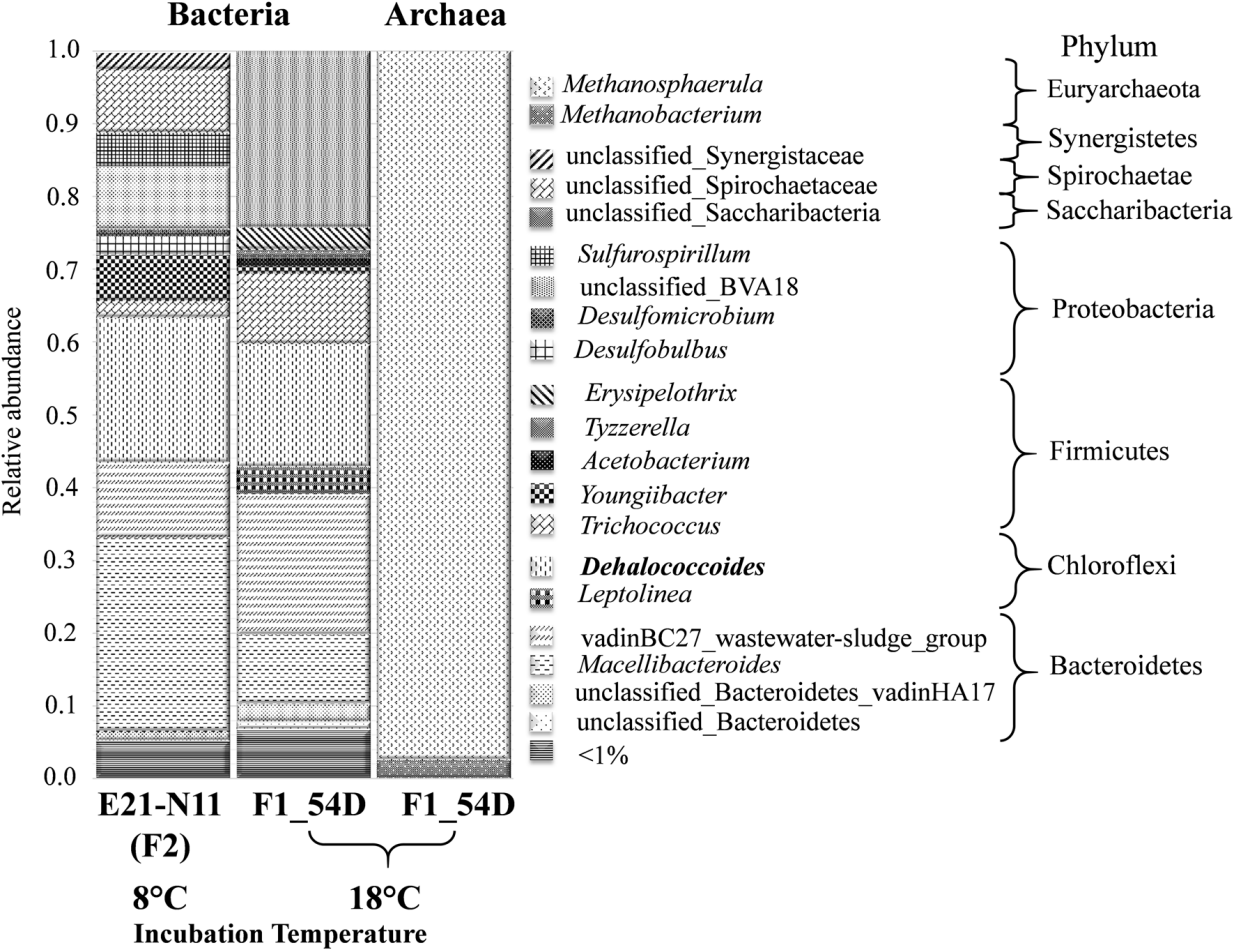
Percentile distribution of amplicon sequences on order and phylum level revealed from fermenter F1 and composite sample of enrichments E21 and N11 used for inoculation of fermenter F2

16S rRNA gene amplicon sequencing of the fermenter F1 and the composite sample of enrichments E21 and N11 used for inoculation of the fermenter F2 resulted in 8650 and 1492 high-quality reads, respectively, classified within the Bacteria domain. Rarefaction curves completely reached a plateau suggesting that the sequencing depth was appropriate to cover the bacterial diversity (data not shown). The diversity estimators including ACE (F1: 106; E21-N11: 42), Chao1 (F1: 106; E21-N11: 42), Shannon–Wiener (F1: 2.4; E21-N11: 2.51) and Simpson’s (F1: 0.14; E21-N11: 0.12) indices implied low diversity of the bacterial community. Common members of the enrichments belonged to the phyla Bacteroidetes, Chloroflexi, Proteobacteria, Firmicutes, Spirochaetae and Synergistetes (Fig. 4). Bacteroidetes and Chloroflexi populations were the most dominant members of both cultures. Significant prevalence of phylum Saccharibacteria was also observed in the bacterial community of the fermenter F1. Representatives of phyla Spirochaetae and Synergistetes were solely identified from the composite enrichment (E21–N11) used for the inoculation of fermenter F2. The organohalide-respiring genus *Dehalococcoides* with relative abundance of 19.83% and 16.98% gave the majority of the microbial community of composite enrichment and fermenter F1, respectively (Fig. 4). In the composite enrichment (E21–N11) other putative dechlorinators as *Sulfurospirillum* sp. and *Geobacter* sp. were present with low relative abundance of 4.5 and < 1%, respectively (Fig. 4). *Sulfurospirillum* sp.*, Geobacter* sp. and *Dehalobacterium* sp. as putative dechlorinators were also detected in the fermenter F1, however, their relative abundance did not exceed the 1%. In addition to the genus *Dehalococcoides* other four abundant OTUs belonging to mainly presumed fermentative (*Macellibacteroides* spp.*, Trichococcus* spp.*, Youngiibacter* spp.) and amino-acid degrading bacteria (vadinBC_27_wastewater-sludge_group) were shared among the two enrichments (Fig. 4). *Dehalocococcides* sp. and the fermentative bacteria represented the core bacterial community of both enrichments. Presumed acetogenic bacteria (*Acetobacterium* spp., *Tyzzerella* spp., *Desulfobacter* spp.*, Desulfobulbus* spp.*, Desulfomicrobium* spp. and *Desulfuromonadales*) with considerably different relative abundances (Fig. 4) were also detected in the enrichments. However, the composition of acetogenic species differed in the two enrichments. Among the acetogens a variety of sulphate-reducing species were detected. According to the Archaea 16S rRNA gene amplicon sequencing of the fermenter F1 *Methanobacterium* and *Methanosphaerula* were identified with the significant prevalence of *Methanosphaerula* (relative abundance 97%) (Fig. 4).

### In silico analysis

Based on the in silico 16S T-RFLP, predicted T-RF sizes corresponded to *Dehalococcoides* sp., *Geobacter* spp. and *Sulfurospirillum* spp. restricted by *Alu*I and *Bsh*1236I. In silico determined base pair length of the individual T-RF’s are consistent with the actual T-RF size and previous results described by Mészáros et al. (2013). Based on the in silico data, the ratio of the *Dehalococcoides* sp. was usually above 5% in the bacterial community of the enrichments characterized with the presence of VC and ethene. The relative abundance of the genus *Dehalococcoides* (up to 18%) showed significant but slow increase in the series of enrichments and the fermenters. Partial TCE degradation to *cis*-DCE was observed in enrichments characterized with the presence of *Sulfurospirillum* spp. (relative abundance up to 4.7%) and/or *Geobacter* spp. (relative abundance up to 29%). However, the co-occurrence of the *Sulfurospirillum* spp. or *Geobacter* spp. was rarely detected.

## Discussion

In our work two sites contaminated with short-chain chlorinated ethenes were selected to enrich organohalide-respiring microbial communities to produce site specific bioactive inocula at industrial scale. Groundwater samples originated from the selected contaminated sites were characterized with autochthonous organohalide-respiring microbial communities capable of complete dechlorination of PCE and TCE to harmless end-product ethene. Despite the presence of the *Dehalococcoides* sp. communities and of the three examined *RDase* genes, TCE was only partially dechlorinated to *cis*-DCE at the non-treated site II suggesting the lack of nutrients for organohalide-respiring microorganisms. While at site I, treated with organic substrate, complete dechlorination of TCE to ethene was observed. Beyond the presence of organohalide-respiring microorganisms, available electron donors and nutrients are also needed for the complete dechlorination (Löffler and Edwards 2006). According to the presence and the activity of the organohalide-respiring bacteria, the collected groundwater samples proved to be appropriate to develop site-specific inocula. Effective dechlorinating microbial consortia were successfully enriched from groundwater samples collected from non-treated and biostimulated sites. The lag phase prior to detectable dechlorination activity in enrichments incubated at 8 °C in 500 mL volume and inoculated with microbial communities derived from different groundwater was usually observed within 30 days independently from their origin. Similar dechlorination performances with comparable time-scale were observed so far at higher temperature, above 12 °C (Cichocka et al. 2010; Löffler et al. 2013b; Mészáros et al. 2013). After the second and third transfer of the enrichments into higher (2 L, 5 L, 10 L) volumes along with TCE addition, the time-course shortened with a progressively accelerated ability of dechlorination. During the serial transfer steps, enrichments inoculated with stimulated organohalide-respiring communities resulted in more extent and rapid dechlorination of TCE to *cis*-DCE compared to the enrichments inoculated with microbial community originated from the non-treated area. The applied biostimulation sufficiently enhanced the organohalide-respiring microorganisms and might have contributed to the accelerated dechlorination and the differently structured microbial communities in enrichments. Beyond the application of biostimulated microbial communities, the enhancement of microbial activity by sequential nutrient and vitamin addition (Mészáros et al. 2013) contributed to the efficient transfer from bench-scale to industrial-scale within less 18 months.

According to our results, *Dehalococcoides* sp. prevailing in the groundwater sustained in the enrichments even in those without ethene production. Complete dechlorination with the presence of VC and ethene was observed in enrichments characterized with the relative abundance of the *Dehalococcoides* sp. exceeding 5%. In line with the accelerated transformation of the TCE, the relative abundance of *Dehalococcoides* sp. increased in the enrichments and the fermenters. Lee and Lee (2016) also found that the relative abundance of the *Dehalococcoides* sp. increased during the serial transfer of dechlorinating consortia. Moreover, they found that the complete dechlorination with ~ 1% relative abundance of *Dehalococcoides* sp. corresponded to 10^8^ 16S rRNA gene copy g^−1^ (Lee and Lee 2016). In our enrichments, especially in fermenters, the total population of *Dehalococcoides* could be assumed equal or higher compared to Lee and Lee’s results (2016). Considering the bioremediation of chlorinated ethenes contamination by bioaugmentation agents Lu et al. (2006) concluded that 10^7^*Dehalococcoides* cells L^−1^ is necessary for the effective dechlorination. However, to achieve appropriate cell density on contaminated sites, up to several hundred litres of bioaugmentation agents should be used (Ellis et al. 2000; Vainberg et al. 2009; Pérez-de-Mora et al. 2014) depending on the *Dehalococcoides* cell density and the size of the contaminated field.

According to the sequence analyses, different *Dehalococcoides* ribotypes affiliated with the Pinellas and Cornell subgroups were sustained in the enrichments differing regarding to electron acceptor utilization. The members of the Pinellas subgroup show highly similar 16S rRNA gene sequences with 1–3 nucleotide differences (Löffler et al. 2013a). To confirm the presence of the different *Dehalococcoides* sp. strains 16S rRNA gene sequencing was supplemented with the phylogenetic information of the *RDase* genes. The co-presence of the three reductive dehalogenase genes suggests at least three different *Dehalococcoides* sp. strains in the fermenters and several enrichments. The different composition and ratio of the *Dehalococcoides* sp. strains could affect the biodegradation rate of the chlorinated ethenes that may have contributed to the observed differences in the dechlorination activity in fermenters. The accelerated dechlorination process without the accumulation of intermediates DCE and VC suggests the predominant presence of *Dehalococcoides* sp. BAV1 strain like microorganism (Cichocka et al. 2010) in the fermenter F2. The incomplete dechlorination to ethene with the periodic detection of the *trans*-DCE in the enrichment of fermenter F1 strengthens the presence of *Dehalococcoides* sp. CBDB1 strain like microorganism (Adrian et al. 2007). Besides the presence of the *Dehalococcoides* sp. CBDB1 strain like microorganism the dechlorination of *trans*-DCE without the accumulation of VC suggests the low ratio of the *Dehalococcoides* BAV1 strain like microorganism detected based on the catabolic *bvcA* gene in the fermenter F1. With the presence of the *Dehalococcoides* sp. CBDB1 and BTF08 strains like microorganisms (Marco-Urrea et al. 2011; Cichocka et al. 2010) and *Sulfurospirillum* sp. (Luijten et al. 2003) in the fermenters F1 and F2, respectively, PCE dechlorination capability could be assumed in the enrichments of the fermenters. The revealed complex and diverse population of the different *Dehalococcoides* sp. strains resulted in the fastest TCE reduction to ethene in the enrichments of the different fermenters, which could be accelerated by the elevated temperature as well (Friis et al. 2007). However, based on the results of amplicon sequencing, the relative abundance of the *Dehalococcoides* sp. in the fermenter F1 was not higher than in composite enrichments used for inoculation of fermenter F2 incubated at 8 °C. Considering the selective effect of the temperature, the presence and the relative abundance of the dominant members as Chloroflexi, Bacteroidetes and Firmicutes in enrichments incubated at different temperature showed similarities. Moreover, Bacteroidetes and Firmicutes were frequently detected in non-dechlorinating community of *Dehalococcoides* sp. dominated consortia incubated at temperature above 20 °C (Ismaeil et al. 2017, 2018; Hug et al. 2012). Furthermore, the composite consortium (E21-N11) incubated at 8 °C exhibited significant enrichment in δ- and ε-Proteobacteria, Spirochaetae and Synergistetes showing similarities to medium DonnaII incubated at 30 °C (Hug et al. 2012). However, based on genus level, discrepancies were observed considering several fermentative bacteria. *Trichococcus* spp. (Liu et al. 2002), *Youngiibacter* spp. (Lawson et al. 2014) and *Macellibacteroides* spp. (Jabari et al. 2012) characterized with high relative abundance were present in the enrichment of the fermenter F1 and presumably originated from the sour whey. According to the presence and pronounced relative abundance of Saccharibacteria, the community composition of the enrichment in the fermenter F1 differed from that of formerly developed cultures (Duhamel et al. 2002; Waller et al. 2005; Duhamel and Edwards 2006; Holmes et al. 2006; Hug et al. 2012). Beside the different origin of the microbial community, the temperature and the surface provided by the solid phase, affected the settlement and the growth of microorganism *inter alia* the *Dehalococcoides* sp. strains. Overall, these parameters had effect on the composition of the organohalide-respiring microbial communities.

Fermentative bacteria can produce lactate, ethanol, hydrogen and acetate used by *Dehalococcoides* sp. as electron donors and carbon source, respectively. Lactate, amended in the enrichments by sour whey, can be transformed to acetate and hydrogen by *Trichococcus* sp. (Révész et al. 2006) and/or can be utilized as a carbon source and electron donor by sulphate-reducing bacteria as well. Similarly, to the observation of Révész et al. (2006) black precipitate was formed in the bottles indicating sulphide production that is indirect evidence for the activity of sulphate-reducing bacteria. The majority of the detected sulphate-reducing bacteria were acetogens implying intrinsic acetate production in the enrichments based on lactate and amino-acids. Fermentation products such as lactate, acetate and hydrogen support the growth of methanogenic Archaea beyond the organohalide-respiring microorganisms. Two members of the domain Archaea were identified with the significant prevalence of the hydrogenotrophic *Methanosphaerula*. To our knowledge this is the first time to detect the presence of *Methanosphaerula* in dechlorinating enrichments which are usually dominated by hydrogenotrophic *Methanomicrobiales, Methanobacteriales,* and acetotrophic *Methanosarcinaceae* and *Methanoseataceae* (Hug et al. 2012). Methanogenesis with increasing methane concentration co-occurred especially with accelerated dechlorination in the enrichment. The decreasing concentration of TCE and its intermediates may have contributed to the increase in methanogenesis, since PCE and its intermediate products such as TCE has inhibitory effect on methanogenic Archaea (DiStefano et al. 1991). However, several studies reported that dechlorination efficiency decreased in line with the accelerated methanogenesis (Aulenta et al. 2005; Men et al. 2013), since methanogens have a significant influence on the composition of the organohalide-respiring microbial communities (Men et al. 2013). According to our results the accelerated methanogenesis had no inhibitory effect on the dechlorination process. Presumably, hydrogen, lactate and acetate could be available in excess due to their addition and intrinsic formation by sour whey protein and biomass decay supporting the growth of dechlorinators and methanogens as well. Probably the availability of alternative electron donor such as lactate for dechlorinators contributed to the reduced competition between the organohalide-respiring and hydrogenotroph methanogen microorganisms. Methanogenic Archaea (Yan et al. 2013) along with the detected *Geobacter* spp., *Sulfurospirillum* spp., *Desulfovibrio* spp., *Acetobacterium* spp. and *Clostridium* spp. can produce corrinoid (Men et al. 2013) which is a preferred cofactor for *Dehalococcoides* sp. strains (Yan et al. 2013) growing on chlorinated ethenes. Similarly, developed enrichments maintained the metabolic functions important in supporting the *Dehalococcoides* sp. growth. This conserved metabolic profile with the presence of fermentative, acetogenic and methanogenic species contributed to complete and elevated dechlorination, which was maintained under low and high temperature as well. According to the dechlorination efficiency and the presence of the *Dehalococcoides* sp., the dechlorinating consortia maintained in fermenters of 100 L volume are suitable for performing pilot-scale tests conducted on site of the origin of organohalide-respiring communities.

## Conclusion

Organohalide-respiring microbial communities were successfully enriched at laboratory- and industrial-scale fermenters. Microbial consortia derived from contaminated sites were used as seed culture characterized with differently structured autochthonous organohalide-respiring communities influenced by the availability of electron donors and nutrients. Nutrient addition on site and during cultivation (sequential transfer of enrichments into fresh media) enhanced the dechlorination process. Through serial transfer processes the detected diversity of *Dehalococcoides* sp. and its related *RDase* genes (*tceA, vcrA* and *bvcA*) altered affecting the progress of dechlorination. To our knowledge this is the first attempt to enrich organohalide-respiring bacteria with accelerated dechlorination along with the increasing relative abundance of *Dehalococcoides* sp. in enrichments amended with clay mineral in pilot scale (upto 100 L) at temperature below 20 °C (8 °C and 18 °C). Our results present cold adapted organohalide-respiring bacterial communities. In addition to the low temperature, the different origin of the microbial community and the solid-phase provided surface could contribute to the unique microbial composition of the developed consortia.

## Electronic supplementary material

Below is the link to the electronic supplementary material.

Supplementary file1 (TIF 347 kb)
Table S1. PCR primer sets and the applied annealing temperatures to detect organohalide-respiring microorganisms. * modified
