# Supplementary figures and images for: Preparation and characterization of site-specific dechlorinating microbial inocula capable of complete dechlorination enriched in anaerobic microcosms amended with clay mineral
Source: World J Microbiol Biotechnol. 2020 Feb 3;36(2):29. doi: 10.1007/s11274-020-2806-7 (PMC6997268; doi:10.1007/s11274-020-2806-7)

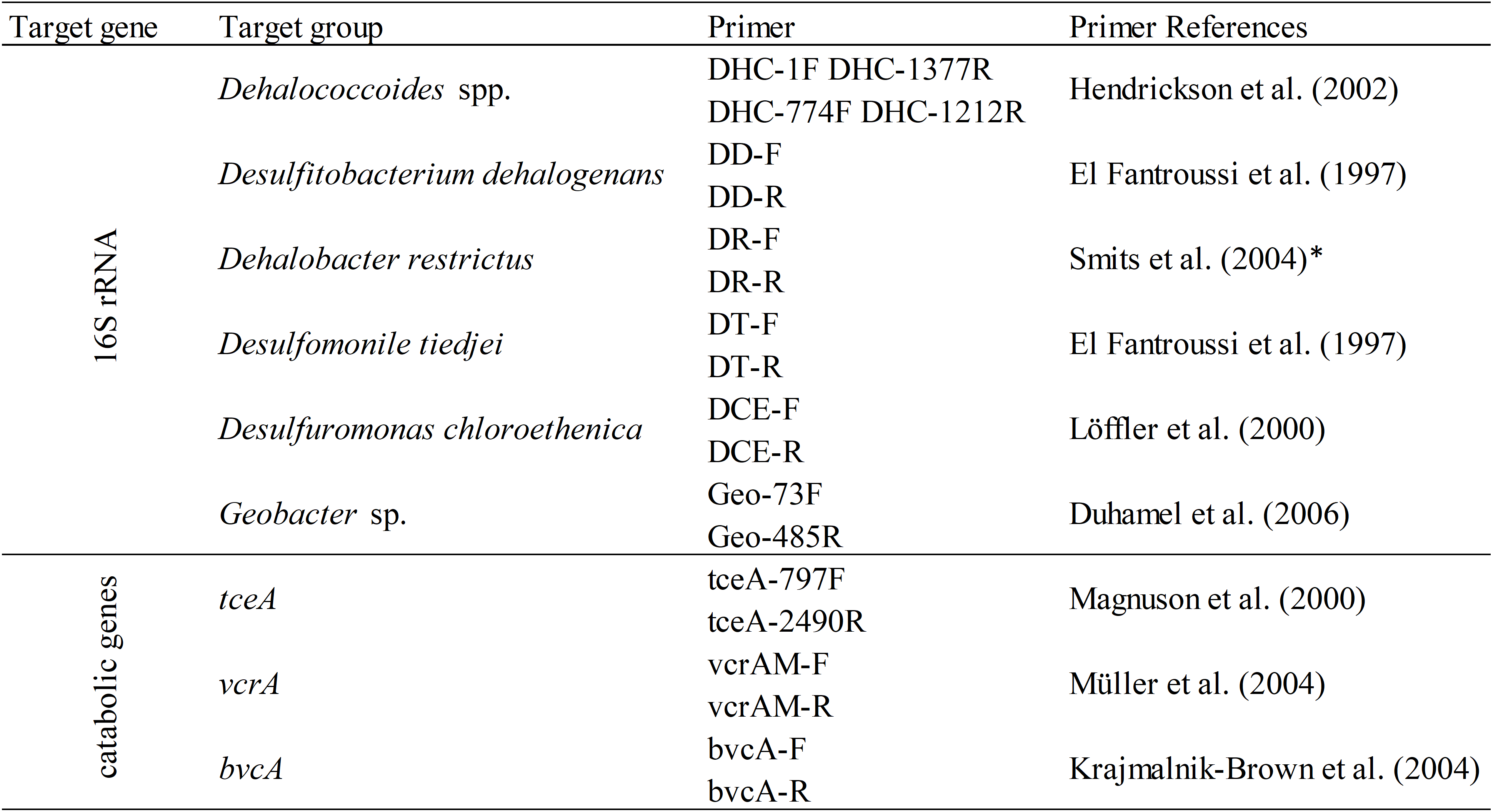

Supplement: Supplementary file 1 — Supplementary file1 (TIF 347 kb) Table S1. PCR primer sets and the applied annealing temperatures to detect organohalide-respiring microorganisms. * modified [file 11274_2020_2806_MOESM1_ESM.tif]
